# Supplementary material for: Tissue-Specific Hormone Signalling and Defence Gene Induction in an In Vitro Assembly of the Rapeseed Verticillium Pathosystem
Source: Int J Mol Sci. 2023 Jun 22;24(13):10489. doi: 10.3390/ijms241310489 (PMC10341851; doi:10.3390/ijms241310489)
Supplement: Supplementary file 1 [file ijms-24-10489-s001.zip › ijms-2384199-supplementary.pdf]

Supplementary Table S1: List of primers used in this study.

Code	Sequence (5′-3′)	Amplicon length (bp)	Annealing Temp.	R^2	Slope	Efficiency	References
OLG70	CAGCGAAACGCGATATGTAG	261	59°C	0.998	-3.232	103.9	Eynck et al. 2007
OLG71	GGCTTGTAGGGGGTTTAGA						
BnaUbiquitin 11-F	AGGCCAAGATCCAAGACAAG	154	60°C	1.0	-3.402	96.8	This study
BnaUbiquitin 11-R	TAGAAACCTCCACGGAGACG						
BnaActin-F	ACGACAGCAGAGCGGGAAAT	188	60°C	0.999	-3.403	96.7	[65]
BnaActin-R	GGCTGGAACAGGACCTCTGG						
BnaTubulin-F	CAGCAATACAGTGCCTTGAGTG	204	60°C	0.999	-3.367	98.2	[66]
BnaTubulin-R	CCTGTGTACCAATGAAGGAAAGCC						
LOX2_F	GTGGGTGCCATCAGAGTTTT	218	60°C	0.999	-3.476	93.95	[67]
LOX2_R	GTCTCCAGCTCCTGTTTTCG						
OPR3_F	GGCAGTGATGAGGAAGAAGC	195	63°C	0.993	-3.467	94.3	This study
OPR3_R	CTTCGCACCAACCTTAAACC						
ACS2_F	AGGTGGTCAAAGACTTAGATAG	128	60°C	0.997	-3.302	100.8	[68]
ACS2_R	ACCGAGTCGTTGTAAGAATA						
ACO4_F	CATTCTACAACCCTGGAAGCGAC	204	59°C	1.0	-3.387	97.3	[69]
ACO4_R	ATGGTCCAACATTGTTGGCCAC						
ICS1_F	ACAGAGTGAAGGGCATGATG	236	60°C	1.0	-3.31	100.5	This study
ICS1_R	TCGAGGCCTAGTTGCAGC						
PR1_F	AAAGCTACGCCGACCGACTACGAG	97	59°C	0.999	-3.265	102.4	[14]
PR1_R	CCAGAAAAGTCGGCGCTACTCCA						

Quantification of *B. napus* defence genes by qRT-PCR

LOX2

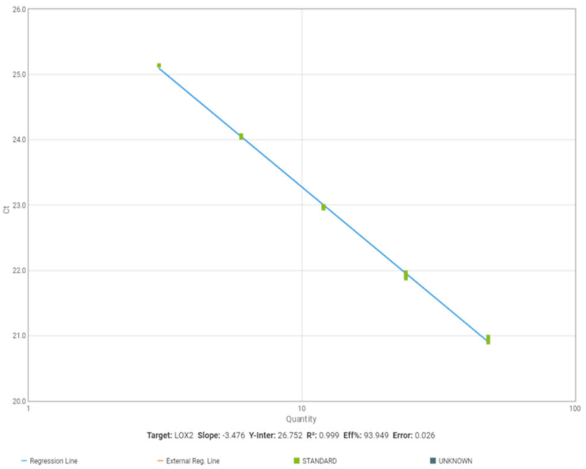

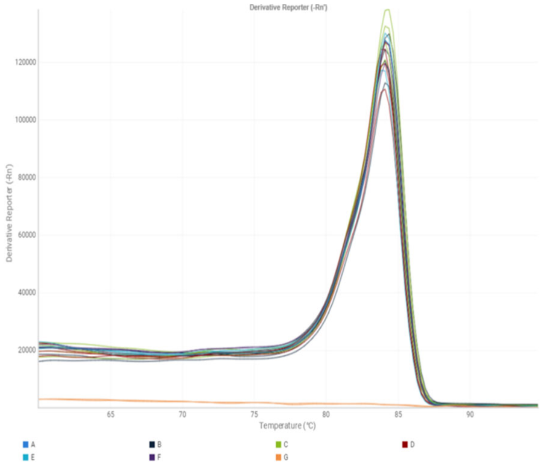

R^2 = 0.999; Primer efficiency = 93.9%

OPR3

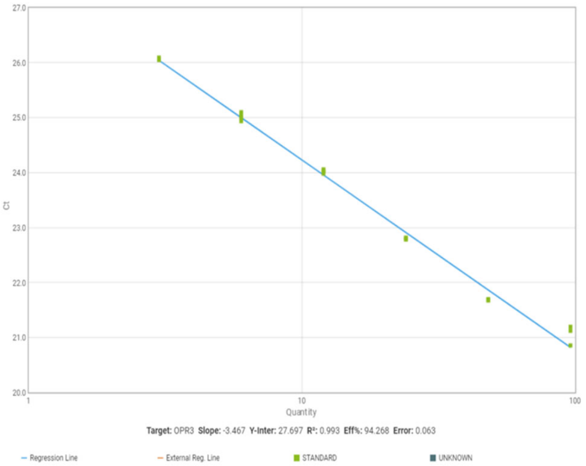

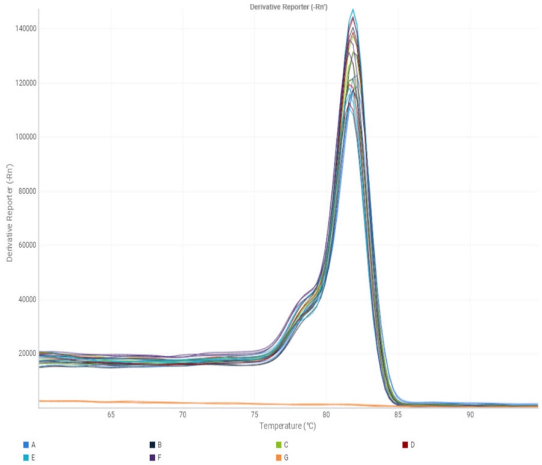

R^2 = 0.993; Primer efficiency = 94.3%

ACS2

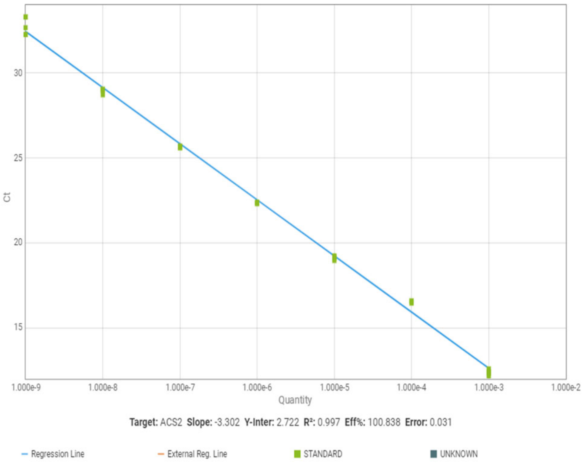

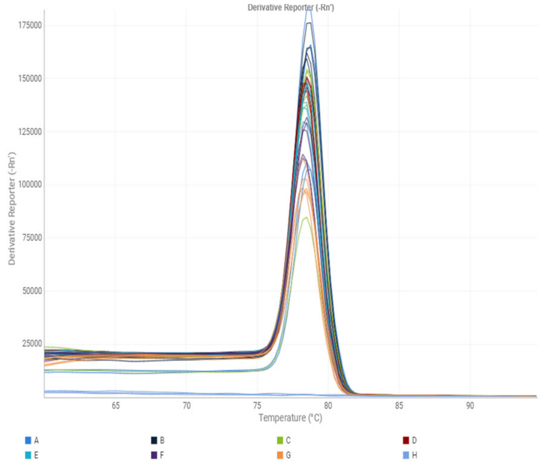

R^2 = 0.997; Primer efficiency = 100.8%

ACO4

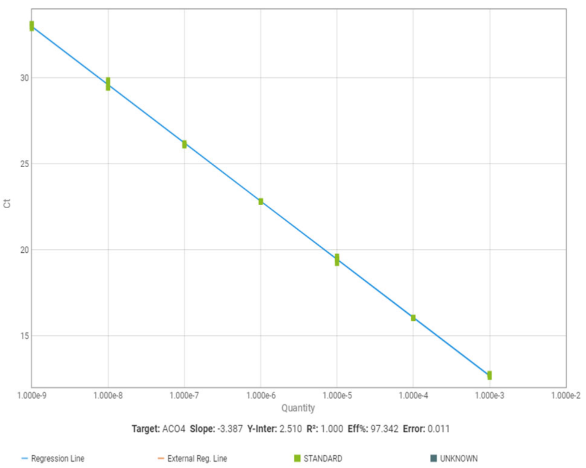

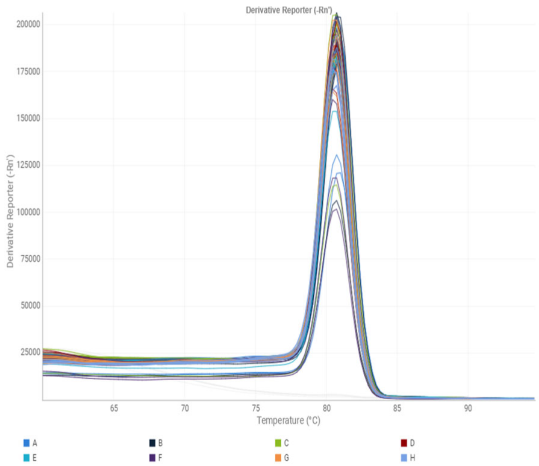

R^2 = 1.000; Primer efficiency = 97.3%

ICS1

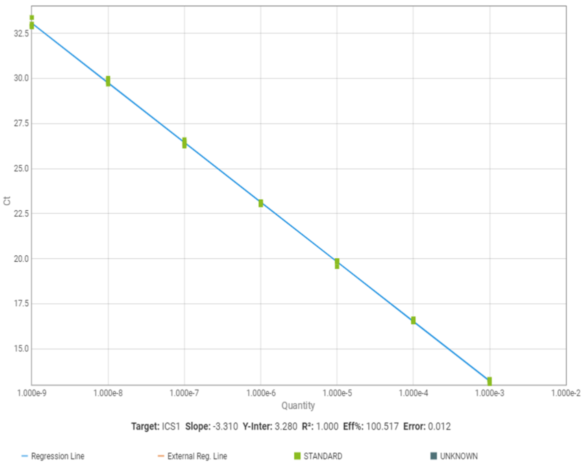

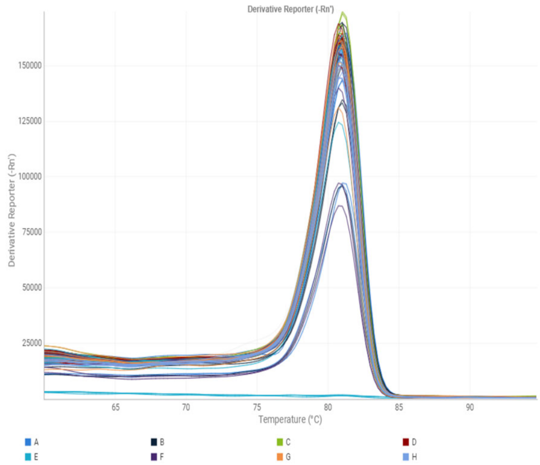

R^2 = 1.000; Primer efficiency = 100.5%

PR1

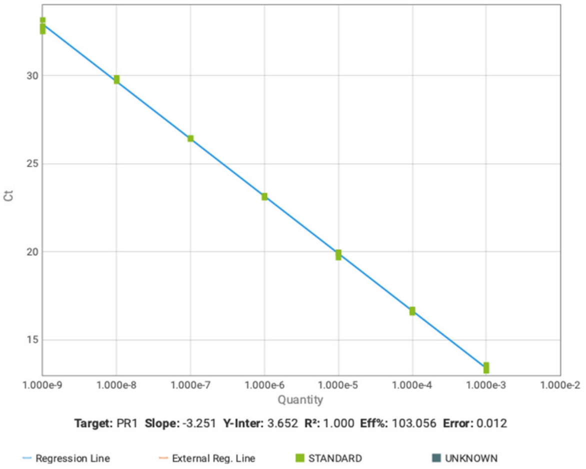

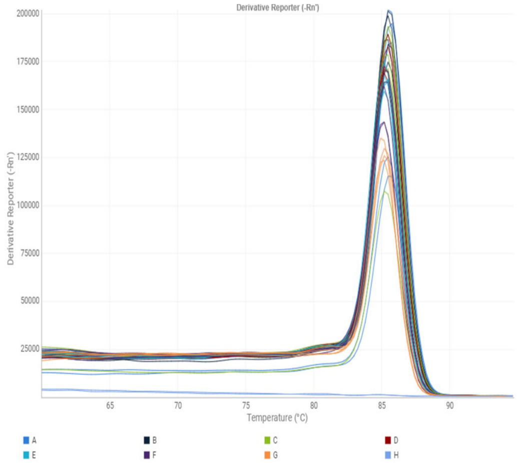

R^2 = 1.000; Primer efficiency = 103.0%

Supplementary Figure S1. Efficiencies of the primer pairs used in this study. Standard curves and melting curves (°C) of resulting PCR products are indicated.

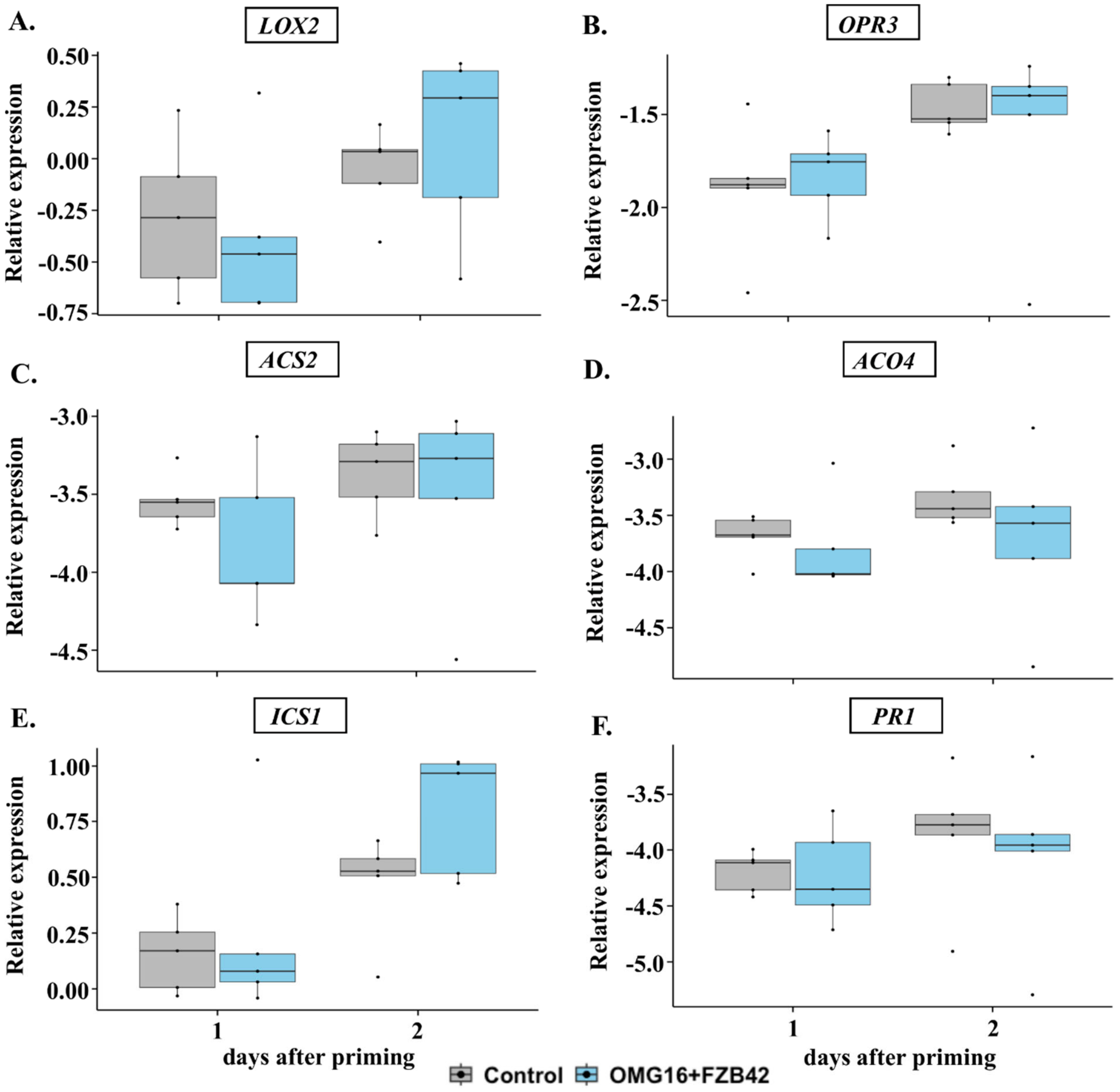

Supplementary Figure S2. Relative expression of rapeseed defence genes *LOX2* (A), *OPR3* (B), *ACS2* (C), *ACO4* (D), *ICS1* (E) and *PR1* (F) in roots upon OMG16 plus FZB42 inoculation prior to Vl43 infection. Roots of two-week-old rapeseed plantlets were primed with OMG16 plus FZB42. The relative transcript abundances of the rapeseed defence genes were analysed by qRT-PCR in roots of the rapeseed plants before Vl43 infection. *BnaUbiquitin11*, *BnaActin* and *BnaTubulin* were used as endogenous controls for normalization. Box and whisker plots show ΔCq values of five biological replicates with quadruplicate qRT-PCRs where significance of gene expression was calculated by unpaired t-test at $P < 0.05$. OMG16, *T. harzianum* OMG16; FZB42, *B. velezensis* FZB42.

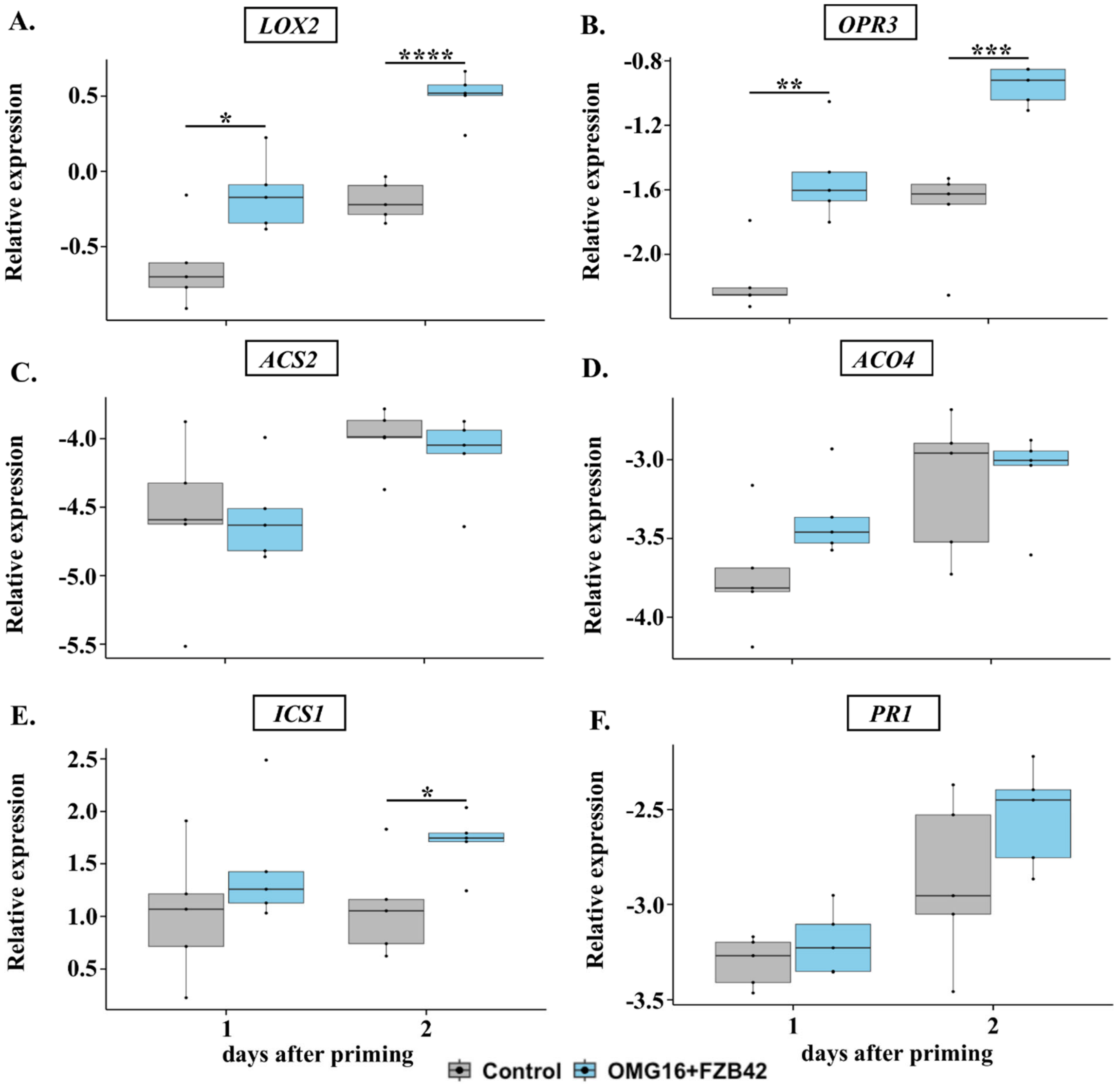

Supplementary Figure S3. Relative expression of rapeseed defence genes *LOX2* (A), *OPR3* (B), *ACS2* (C), *ACO4* (D), *ICS1* (E) and *PR1* (F) in stems upon OMG16 plus FZB42 inoculation prior to Vl43 infection. Roots of two-week-old rapeseed plantlets were primed with OMG16 plus FZB42. To assess systemic responses, the relative transcript abundances of the rapeseed defence genes were analysed by qRT-PCR in stems of the same plants tested in Supplementary Figure S2 before Vl43 infection. *BnaUbiquitin11*, *BnaActin* and *BnaTubulin* were used as endogenous controls for normalization. Box and whisker plots show ΔCq values of five biological replicates with quadruplicate qRT-PCRs where significance of gene expression was calculated by unpaired t-test at $P < 0.05$. * $P < 0.05$; ** $P < 0.01$; *** $P < 0.001$; **** $P < 0.0001$. OMG16, *T. harzianum* OMG16; FZB42, *B. velezensis* FZB42.

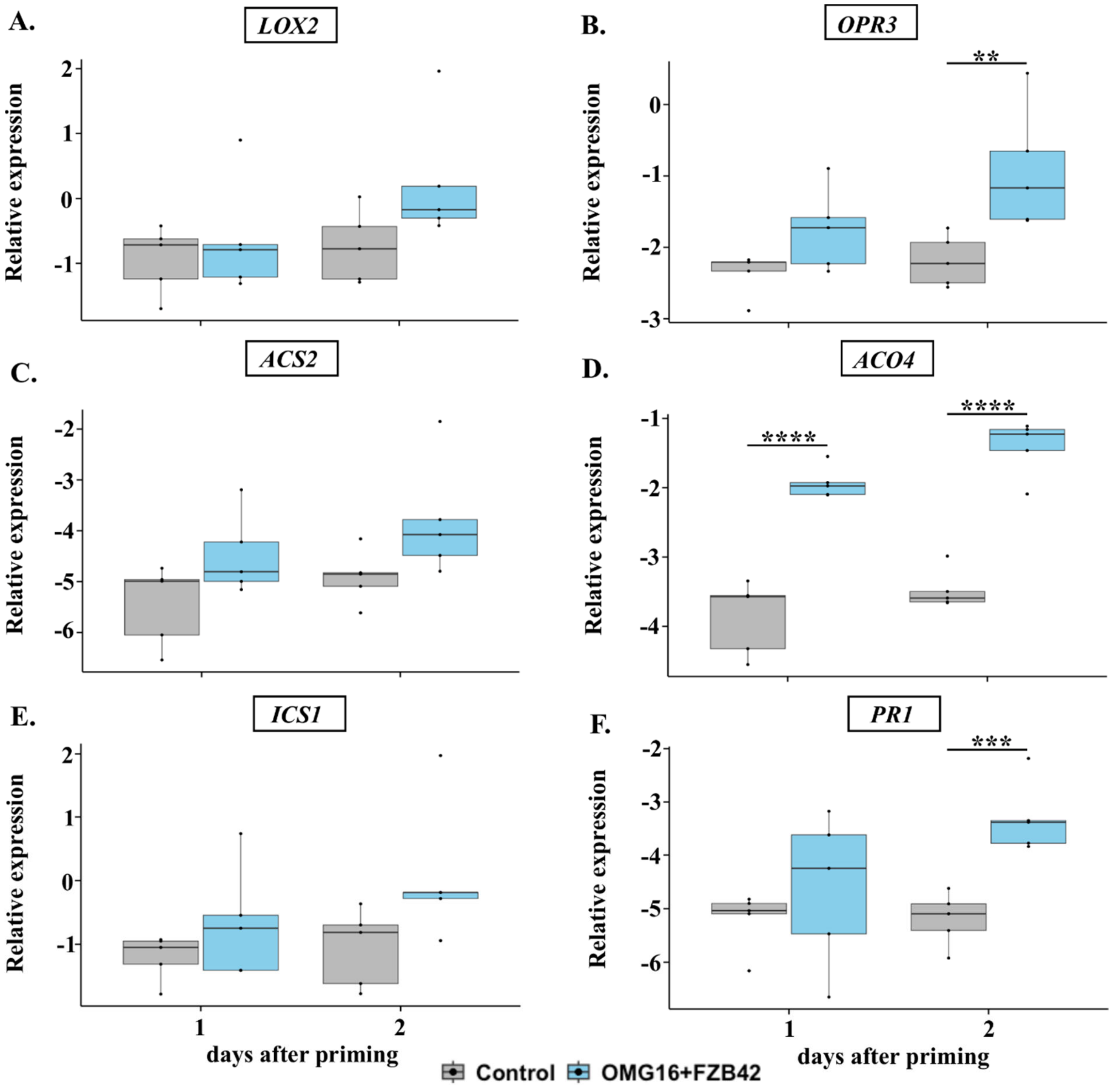

Supplementary Figure S4. Relative expression of rapeseed defence genes *LOX2* (A), *OPR3* (B), *ACS2* (C), *ACO4* (D), *ICS1* (E) and *PR1* (F) in leaves upon OMG16 plus FZB42 inoculation prior to Vl43 infection. Roots of two-week-old rapeseed plantlets were primed with OMG16 plus FZB42. To assess systemic responses, the relative transcript abundances of the rapeseed defence genes were analysed by qRT-PCR in leaves of the same plants tested in Supplementary Figure S2 before Vl43 infection. *BnaUbiquitin11*, *BnaActin* and *BnaTubulin* were used as endogenous controls for normalization. Box and whisker plots show ΔCq values of five biological replicates with quadruplicate qRT-PCRs where significance of gene expression was calculated by unpaired t-test at $P < 0.05$.; ** $P < 0.01$; *** $P < 0.001$; **** $P < 0.0001$. OMG16, *T. harzianum* OMG16; FZB42, *B. velezensis* FZB42.
